# Cognitive functions and patterns of brain activity in patients after simultaneous coronary and carotid artery revascularization

**DOI:** 10.3389/fnhum.2023.996359

**Published:** 2023-04-12

**Authors:** Irina Tarasova, Olga Trubnikova, Darya S. Kupriyanova, Olga Maleva, Irina Syrova, Irina Kukhareva, Anastasia Sosnina, Roman Tarasov, Olga Barbarash

**Affiliations:** ^1^Department of Clinical Cardiology, State Research Institute for Complex Issues of Cardiovascular Diseases, Kemerovo, Russia; ^2^Department of Cardiac and Vascular Surgery, State Research Institute for Complex Issues of Cardiovascular Diseases, Kemerovo, Russia; ^3^Research Institute for Complex Issues of Cardiovascular Diseases, Kemerovo, Russia

**Keywords:** simultaneous revascularization, coronary artery bypass grafting, carotid endarterectomy, postoperative cognitive dysfunction, brain electrical activity

## Abstract

**Background:**

On-pump coronary artery bypass grafting (CABG) is associated with a high risk of neurological complications in patients with severe carotid stenosis. Moreover, early postoperative cognitive dysfunction (POCD) incidence remains high in patients undergoing simultaneous coronary and carotid surgery. Recent studies have shown that even moderate carotid stenosis (≥50%) is associated with postoperative cognitive decline after CABG. Data on brain health in the postoperative period of simultaneous coronary and carotid surgery are limited.

**Objectives:**

This study aimed to analyze early postoperative changes in the cognitive function and patterns of brain electrical activity in patients after simultaneous coronary and carotid artery revascularization.

**Materials and methods:**

Between January 2017 and December 2020, consecutive patients were assigned to on-pump CABG with or without carotid endarterectomy (CEA) according to clinical indications. An extended neuropsychological and electroencephalographic (EEG) assessment was performed before surgery and at 7–10 days after CABG or CABG + CEA.

**Results:**

A total of 100 patients were included [median age 59 (55; 65), 95% men, MMSE 27 (26; 28)], and among these, 46 underwent CEA. POCD was diagnosed in 29 (63.0%) patients with CABG + CEA and in 32 (59.0%) patients with isolated CABG. All patients presented with a postoperative theta power increase. However, patients with CABG + right-sided CEA demonstrated the most pronounced theta power increase compared to patients with isolated CABG.

**Conclusion:**

The findings of our study show that patients with CABG + CEA and isolated CABG have comparable POCD incidence; however, patients with CABG + right-sided CEA presented with lower brain activity.

## 1. Introduction

Patients with high-grade carotid stenosis or occlusion have a high risk of neurological complications during on-pump coronary artery bypass surgery (CABG) (Sahni and Dalton, 2016; Tarasov et al., 2017; Campbell et al., 2021). A possible approach that has been recognized in some cases as an effective treatment modality is CABG and simultaneous carotid endarterectomy (CEA) (Tarasov et al., 2017; Aboyans et al., 2018; Silverman, 2019). The risk of ischemic brain injury increases due to global or local ischemia and the factors associated with simultaneous coronary and carotid surgery. Although this procedure is not always complicated by traumatic brain injury (stroke), it may cause a less pronounced, diffuse lesion that later leads to cognitive decline (Weimar et al., 2017; Maleva et al., 2020). Previous studies have shown that patients undergoing simultaneous coronary and carotid artery surgery present with a high incidence of postoperative cognitive dysfunction (POCD) in the early postoperative period (Maleva et al., 2019, 2020).

Data on the influence of the laterality of CA revascularization on adverse neurological outcomes are limited (Bossema et al., 2007; Baracchini et al., 2012; Heyer et al., 2015). The study by Bossema et al. (2007) demonstrated that cognitive changes, measured by neuropsychological tests sensitive to hemispheric specialization, are irrespective of the side of intervention. Baracchini et al. (2012) also found no impact of the CEA side on any of the indicators of cognitive performance. Heyer et al. (2015) investigated the relationship between the laterality of CEA and fine hand deficits using the Grooved Pegboard test. The authors demonstrated greater subtle deficits of hand coordination in the non-dominant hand compared to the dominant hand in patients undergoing CEA of the opposite carotid artery.

Thus, the issue of selecting the optimal strategy in the surgical treatment of coronary and carotid atherosclerosis and early diagnosis of brain ischemia during simultaneous coronary and carotid artery surgery is far from being solved and necessitates the implementation of highly informative techniques. Electroencephalography (EEG) may be a promising method for obtaining data on specific changes in the brain electrical activity in cardiac surgery patients due to high temporal and moderate spatial resolution. The study of spontaneous electrical activity in the brain in various pathological conditions revealed that neuronal oscillatory systems are widely involved and that such changes are the earliest evidence of subsequent impairment of cognitive functions (Bonanni et al., 2015; Engels et al., 2016; Tarasova et al., 2021). The brain's electrical activity is affected by temperature management during cardiopulmonary bypass, the depth of anesthesia, metabolic disorders, in particular hypo- or hyperglycemia, and impaired cerebral autoregulation (Howard et al., 2012; Sutter et al., 2013). A number of studies have demonstrated a high diagnostic value of EEG parameters in patients undergoing CABG (Tarasova et al., 2021; Trubnikova et al., 2021). EEG theta band activity is one of the most sensitive indicators associated with perioperative brain injury. According to other studies, even moderate and small stenosis (≥50%) is associated with pronounced theta activity changes in the early postoperative period of CABG (Trubnikova et al., 2014).

Researchers have become interested in the topographic features of brain lesions after cardiac surgery. Cerebral hypoperfusion during on-pump cardiac surgery can contribute to the development of mild, multiple lesions in the frontal and parietal brain lobes, the so-called “watershed areas,” in the terminal branches of adjacent large cerebral arteries (Safan et al., 2022). At the same time, it was found that cerebral blood flow alterations in the frontal and parietal lobes are associated with a decrease in attention and executive control (Hshieh et al., 2017; Wang et al., 2022).

Data on the brain electrical activity in the postoperative period of simultaneous interventions on coronary and carotid stenosis are quite limited. Other authors have shown that patients with CABG + left-sided CEA had a localized postoperative theta activity increase at 7–10 days after surgery compared to baseline. At the same time, patients with isolated CABG or CABG + right-sided CEA demonstrated a more diffuse theta activity increase (Tarasova et al., 2019).

The aim of our study was to investigate early postoperative changes in cognitive functions and patterns of brain electrical activity in patients undergoing simultaneous coronary and carotid surgery. Moreover, we aimed to determine the significance of the laterality of the CA revascularization side in simultaneous interventions (CABG + CEA) for regional EEG power changes.

## 2. Materials and methods

### 2.1. Patients

**Figure 1:**
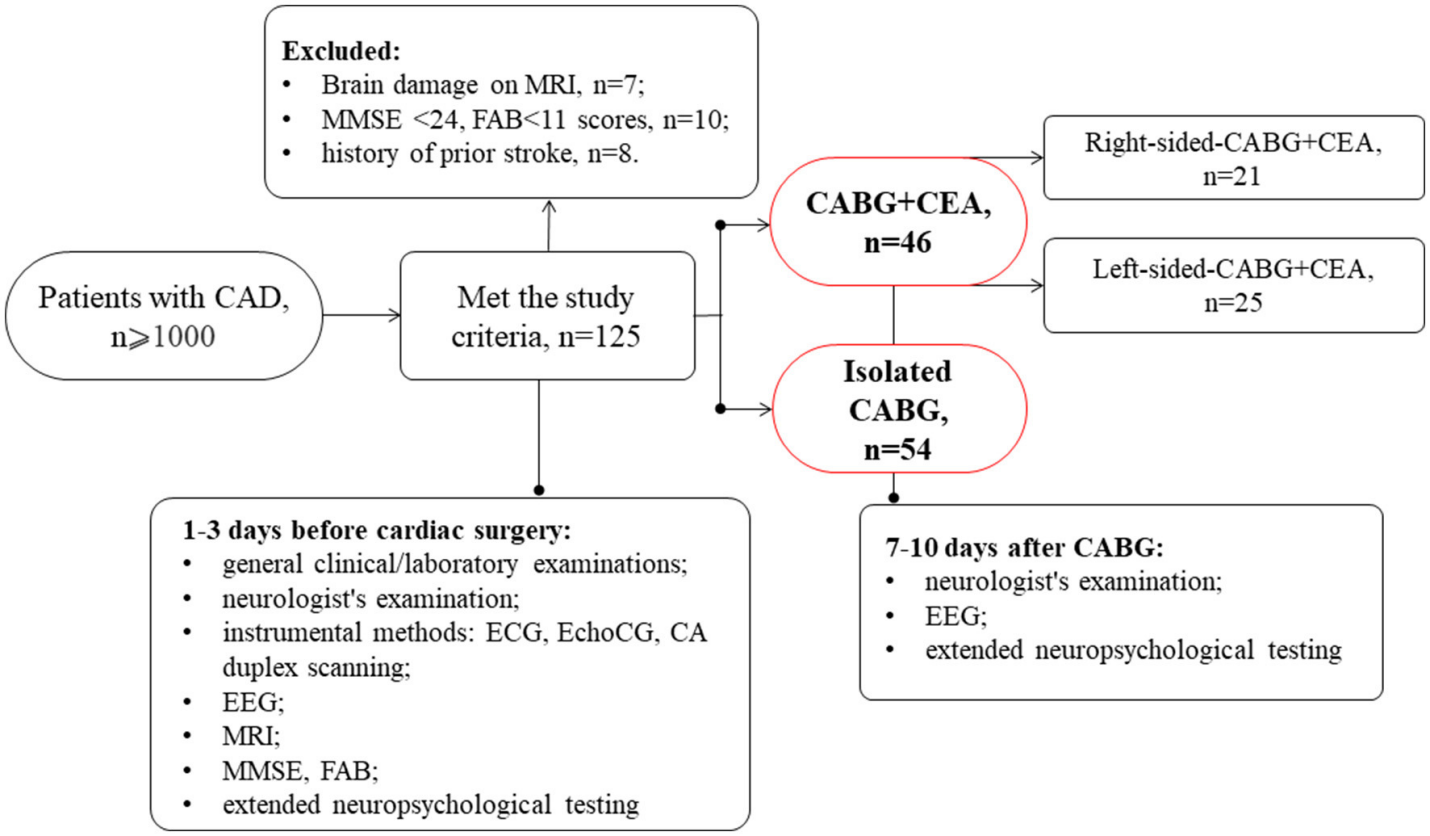
Design of the study.

This prospective and observational study involved 100 patients with indications for simultaneous coronary and carotid artery surgery or isolated CABG selected from a cohort of cardiac patients admitted to the clinic of the Research Institute of Complex Issues of Cardiovascular Diseases. Data collection was performed between January 2017 and December 2020, and consecutive patients were assigned to on-pump CABG with or without simultaneous carotid endarterectomy (CEA) according to clinical indications. This study was performed in compliance with the ethical principles of the Declaration of Helsinki. The study protocol received approval from the Institutional Review Board (study protocol No 2/02072019). All patients included in the study signed an informed consent form (Figure 1).

The inclusion criteria were as follows: patients who were aged between 45 and 80 years, patients with simultaneous coronary and carotid artery revascularization or isolated CABG, and patients who were right-handed (to exclude any influence of the laterality on cognitive function).

The exclusion criteria were as follows:

- Life-threatening arrhythmias (at baseline);
- Functional class IV heart failure according to the New York Heart Association (FC NYHA IV) guidelines;
- Chronic obstructive pulmonary disease with persistent breathing difficulty;
- Malignant pathology;
- Diseases of the central nervous system, including stroke;
- Depressive symptoms [Beck Depression Inventory (BDI-II) score ≥ 8];
- Mini-Mental State Examination (MMSE) score <24; Frontal Assessment Battery (FAB) score < 11; and
- Drugs and alcohol addiction.

The patients underwent standard physical, instrumental, and neurological examinations. The clinicians were blind to patients' study participation.

A total of 46 patients had hemodynamically significant CA stenosis (NASCET criteria) confirmed by digital angiography. The patients with simultaneous coronary and carotid surgery were divided into groups depending on the CEA side: the CABG + left-sided CEA group included 25 patients and the CABG + right-sided CEA group included 21 patients. The group of patients with isolated CABG included 54 patients.

As seen in Table 1, clinical and anamnestic characteristics of patients before surgery were comparable for most indicators. It should be noted that patients with CABG + right-sided CEA were older and had lower MMSE scores. Moreover, the parameters of the intraoperative period such as the mean cardiopulmonary bypass (CPB) time and aorta cross-clamping time were higher in the isolated CABG group compared to the simultaneous intervention groups.

**Table 1:** Clinical and anamnestic characteristics of the groups of the patients before cardiac surgery.

**Variable**	**Isolated CABG *n =* 54**	**CABG + left-sided CEA *n =* 25**	**CABG + right-sided CEA *n =* 21**	***p-*value**
	**1**	**2**	**3**	
Age, years, Me (Q25; Q75)	58.5 (51.5;60.5)	58.5 (56.0;65.0)	69.0 (62.0;74.0)	p1–3 ≤ 0.0001
Mini–mental state, scores, Me (Q25; Q75)	28.0 (27.0;28.0)	27.0 (26.0;28.0)	26.0 (25.0;27.0)	p1–3 = 0.01
Frontal assessment battery, scores, Me (Q25; Q75)	16.0 (16.0;17.0)	16 (16.0;17.0)	15.0 (14.0;17.0)	n/s
BDI–II, scores, Me (Q25; Q75)	2.5 (2.0;4.0)	2.0 (1;4.0)	3.0 (2.0;8.0)	n/s
Educational attainment, years, *n* (%)				
8–14	37 (69)	19 (76)	18 (85)	n/s
≥15	17 (31)	6 (24)	3 (15)	
Functional class of angina, *n* (%)				
I–II	28 (52)	21 (84)	17 (81)	p1–2 = 0.025
III	26 (48)	4 (16)	4 (19)	p1–3 = 0.1
Functional class NYHA, *n* (%)				
I–II	46 (85)	23 (92)	20 (95)	n/s
III	8 (15)	2 (8)	1 (5)	
History of myocardial infarction, *n* (%)	46 (85)	17 (68)	16 (76)	n/s
LVEF, %, Me (Q25; Q75)	56.0 (52.0; 62.0)	57.0 (46.0; 67.0)	65.0 (60.0; 68.0)	n/s
Type 2 of diabetes mellitus, *n* (%)	14 (26)	9 (36)	9 (43)	n/s
History of hypertension, *n* (%)	52 (96)	24 (96)	20 (95)	n/s
Hyperlipidaemia, *n* (%)	38 (70)	24 (96)	17 (81)	n/s
Degree of operated CS, %	-	80.0 (55.0; 99.0)	80.5 (57.0; 99.0)	n/s
Patients with significant contralateral CS, *n* (%)	-	17 (68)	16 (76)	n/s
Cardiopulmonary bypass time, min, Me (Q25; Q75)	106.0 (100.0; 111.0)	79.0 (51.0; 137.0)	86.0 (52.0; 146.0)	p1–2 = 0.01
				p1–3 = 0.02
Aorta cross-clamping time, min, Me (Q25; Q75)	58.0 (50.0; 66.0)	50.0 (28.0; 75.0)	51.0 (27.0; 80.0)	p1–2 = 0.02
				p1–3 = 0.03
CEA time	-	25.0 (20.0; 25.0)	26.0 (21.0; 30.0)	n/s
Total surgery/anesthesia time	162.0 (140.0; 190.0)	90.0 (60.0; 170.0)	90.0 (62.0; 161.0)	p1–2 = 0.01
				p1–3 = 0.01

BDI-II, Beck Depression Inventory; NYHA, heart failure by the New York Heart Association; LVEF, left ventricle ejection fraction; CS, carotid stenosis; CEA, carotid endarterectomy.

### 2.2. Neurophysiological assessment

Cognitive screening tests were performed once at baseline (1–3 days before surgery) in all patients using modified Russian versions of the MMSE and FAB scales. The extended neuropsychological testing and EEG recording were conducted at baseline (1–3 days before surgery) and 7–10 days after surgery.

#### 2.2.1. The neuropsychological test battery

The extended neuropsychological test battery from psychophysiological software ≪Status PF≫ (Ivanov et al., 2001) was used to assess three domains of cognitive function (psychomotor speed and executive function, attention, and short-term memory). Psychomotor speed and executive functions were evaluated using the complex visual-motor response time test, neural responses to feedback, and brain responses to feedback assessments. Bourdon's test was used to assess attention. The visual short-term memory assessment consisted of tasks requiring participants to memorize 10 words, 10 numbers, and 10 non-sense syllables. A detailed description of the neuropsychological test battery is presented in Table 2 (Trubnikova et al., 2021). Alternative versions of the neuropsychological tests were used in repeated measurements to minimize practice effects. Postoperative changes in cognitive function were assessed individually in each patient. The percentage of change in indicators was calculated using the formula: [(baseline value–postoperative value)/baseline value] × 100%. A 20% decline in postoperative parameters compared to baseline in 20% of the test battery indicates POCD (Trubnikova et al., 2021).

**Table 2:** Cognitive test battery for assessing cognitive function in cardiac surgery patients.

**Cognitive tests and indicators**	**Description of the procedure**
**Mini-mental state examination (MMSE)** Scores	30-point questionnaire for cognitive impairment and dementia screening.
**Frontal assessment battery (FAB)** Scores	18-point questionnaire for frontal lobes dementia screening.
**Complex visual-motor reaction** Reaction time, ms Errors, *n*	Reaction latencies of the right and left hands to stimuli (different colors of rectangles) when the subject should choose one of the three presented signals (the number of signals in the test is 30).
**Level of functional mobility of nervous processes responses to feedback**Reaction time, ms Errors, *n* Missed signals, *n*	The previous test is conducted in the feedback mode. The duration of the exposure to the test signal (see above) is changed automatically; the exposure of the next signal is shortened by 20 ms with each correct answer and extended by 20 ms, if the answer is wrong (the number of signals in the test is 120). No response to the appearance of the test signal indicates missed signals.
**Performance of the brain responses to feedback** Reaction time, ms Errors, *n* Missed signals, *n*	The previous test is conducted in the feedback mode for a fixed period of time (5 min). It is necessary to process the maximum number of signals presented with a given exposure.
**The Bourdon's test** Processed letters per 1th min, *n* Processed letters per 4th min, *n*	The subject is provided with the alphabetic version of the Bourdon's test to highlight certain letters for the time of 4 mins.
**10 words memorizing test**, *n*	To remember as many of 10 words presented one after another as possible.
**10 numbers memorizing test**, *n*	To remember as many of 10 numbers presented one after another as possible.
**10 nonsense syllable memorizing test**, *n*	To remember as many of 10 nonsense syllables presented one after another as possible.

#### 2.2.2. EEG recording and processing

**Figure 2:**
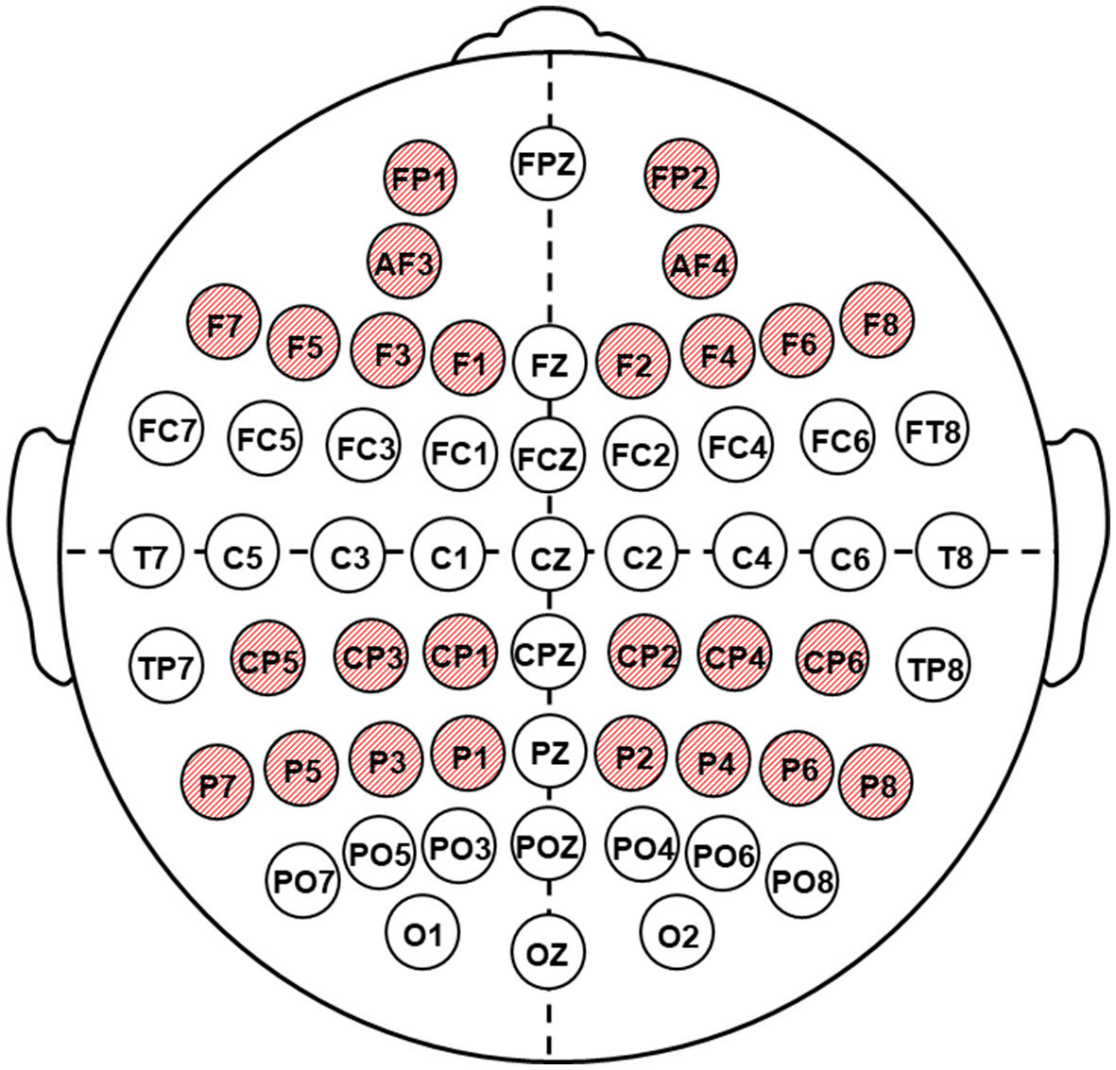
Location of the electrodes of EEG recording. Shaded red circles indicate the regions of interest (ROI).

EEGs were recorded *via* a 62-channel Quik-cap (Neuroscan, El Paso, TX); scalp electrode locations were based on the modified 10/10 system; and a nose bridge electrode was used as a reference. Bipolar eye movement electrodes were applied to the canthus and cheekbone to monitor eye movement artifacts. Electrode impedances were <20 kΩ for all electrodes. The EEGs were recorded using a NEUVO-64 system (NeuroScan, El Paso, TX) in the eyes-closed condition, in a dimly lit, soundproof, electrically shielded room. The EEG recording length was 5 min. The amplifier bandwidths were 1.0 to 50.0 Hz, and EEGs were digitized at 1,000 Hz. Each EEG record was plotted and visually examined and then edited to remove artifacts using the NeuroScan 4.5 software program (Compumedics, TX, USA). Artifact-free EEG fragments were divided into 2s epochs and underwent Fourier transformations. For each subject, EEG power values were averaged within the theta1 (4–6 Hz) range, taking into account the results of previous studies indicating the diagnostic significance of low-frequency rhythm changes in the detection of postoperative ischemic brain injury (Tarasova et al., 2019, 2021). The next step was the clustering of data recorded in 56 leads into five electrode zones symmetrically in the left and right hemispheres: frontal (F) (Fp1/2, AF3/4, F1/2, Fp3/4, Fp5/6, F7/8), central (C) (FC1/2, FC3/4, FC5/6, C1/2, C3/4, and C5/6), temporal (T) (FT7/8, T7/8, and TP7/8), parietal (P) (CP1/2, CP3/4, CP5/6, P1/2, P3/4, P5/6, P7/8), and occipital (O) (PO3/4, PO5/6, PO7/8, O1/2). In the present study, the frontal and parietal zones were considered as the regions of interest (ROI) (Figure 2).

### 2.3. Statistical analysis

The data were analyzed using STATISTICA 10.0 software (StatSoft, Tulsa, OK, USA). The Shapiro–Wilk test was used to test the normality of data. Most of the data (clinical parameters and cognitive indicators) were non-normal; thus, the Mann–Whitney test was used to analyze it. Log-transforming EEG power spectral data were performed in order to normalize the data. Further analysis of the EEG data was done by performing a repeated measure ANOVA. Planned comparisons were used to compare every two tests, a *p*-value of <0.05 was considered statistically significant.

## 3. Results

### 3.1. Neuropsychological functioning

#### 3.1.1. Before cardiac surgery

Executive functions and psychomotor speed, attention, and short-term memory were analyzed, and significant intergroup differences at baseline were noted in the indicators of psychomotor speed and executive function. Increased psychomotor speed was noted in the CABG group, whereas in the CABG + CEA group, this indicator was lower, see Table 3. Moreover, CABG + CEA patients made fewer errors in the executive function test.

**Table 3:** Cognitive indicators in the patients before cardiac surgery.

**Cognitive tests and indicators**	**Isolated CABG *n =* 54**	**CABG+CEA *n =* 46**	**F**	***p* (Mann–Whitney *U* test)**
**Complex visual-motor reaction**				
Reaction time, ms	564.0 [526.0; 620.0]	621.0 [575.0; 736.0]	−3.37	0.0008
Errors, *n*	2.0 [1.0; 3.0]	2.0 [1.0; 3.0]	0.08	0.93
**Level of functional mobility of nervous processes responses to feedback**				
Reaction time, ms	453.0 [424.0; 482.0]	492.5 [453.5; 527.5]	−2.73	0.006
Errors, *n*	25.0 [22; 27]	25 [19; 28]	0.46	0.65
Missed signals, *n*	17 [11; 21]	25 [19; 28]	−1.32	0.19
**Performance of the brain responses to feedback**				
Reaction time, ms	430.0 [401.0; 460]	454.0 [422.0; 486.0]	−1.96	0.049
Errors, *n*	119.0 [111.0; 137.0]	103.0 [89.0; 121.0]	2.75	0.006
Missed signals, *n*	57.0 [40.0; 80.0]	72.0 [45.0; 109.0]	−1.24	0.21
**The Bourdon's test**				
Processed letters per 1 th min, *n*	68.0 [52.0; 83.0]	71.0 [53.0; 89.0]	−0.39	0.70
Processed letters per 4 th min, *n*	88.0 [79.0; 119.0]	94.0 [77.0; 107.0]	−0.04	0.97
**Memory**				
10 numbers memorizing test, *n*	5.0 [4.0; 5.0]	4.0 [3.0; 5.0]	1.12	0.26
10 words memorizing test, *n*	4.0 [4.0; 5.0]	4.0[3.0; 5.0]	1.76	0.08
10 nonsense syllable memorizing test, *n*	2.0 [2.0;4.0]	3.0 [2.0;4.0]	−1.51	0.13

#### 3.1.2. After cardiac surgery

Adverse cardiovascular events (myocardial infarction, stroke, death, and repeated unplanned revascularization) were not observed in the examined patients in the early post-operative period simultaneous with CABG + CEA or isolated CABG.

In our cohort, POCD occurred in 29 (63.0%) patients with CABG + CEA and in 32 (59.0%) patients with isolated CABG (OR = 1.17, 95 % CI = 0.52–2.63, *p* = 0.7). Thus, the incidence of postoperative cognitive deficit was comparable in simultaneous and isolated cardiac surgery.

Postoperative indicators of psychomotor speed and executive function, attention, and short-term memory were analyzed. Significant intergroup differences were detected in the indicators of psychomotor speed and executive function compared to baseline. At 7–10 days after surgery, psychomotor speed was higher in the CABG group compared to the CABG + CEA group in the executive function tests, see Table 4. The isolated CABG patients also presented with higher indicators of executive control function at 7–10 days after surgery compared to patients with CABG + CEA.

**Table 4:** Cognitive indicators in the patients after cardiac surgery.

**Cognitive tests and indicators**	**Isolated CABG *n =* 54**	**CABG+CEA *n =* 46**	**F**	***p* (Mann–Whitney *U* test)**
**Complex visual-motor reaction**				
Reaction time, ms	511.5 [489.0; 557.5]	624.0 [566.0; 679.0]	−4.39	≤ 0.00001
Errors, *n*	2.0 [1.0; 3.0]	2.0 [1.0; 3.0]	0.05	0.96
**Level of functional mobility of nervous processes responses to feedback**				
Reaction time, ms	438.5 [410.0; 461.5]	499.0 [457.5; 516.5]	−3.84	0.00006
Errors, *n*	26.0 [24.0; 30.0]	25.0 [19.5; 28.0]	1.43	0.15
Missed signals, *n*	12.0 [7.5; 16.5]	25.0 [13.5; 28.0]	−3.03	0.002
**Performance of the brain responses to feedback**				
Reaction time, ms	422.0 [403.0; 451.5]	479.0 [430.0; 515.0]	−3.65	0.0003
Errors, *n*	132.0 [113.0; 147.0]	94.0 [76.0; 121.0]	3.27	0.001
Missed signals, *n*	45.0 [20.0; 90.0]	66.0 [50.0;106]	−1.62	0.11
**The Bourdon's test**				
Processed letters per 1 th min, *n*	70.0 [55.0; 91.0]	69.5 [61.5; 92.0]	−0.37	0.97
Processed letters per 4 th min, *n*	98.0 [74.0;111.0]	80.0 [74.0; 100.5]	1.17	0.24
**Memory**				
10 numbers memorizing test, *n*	5.0 [4.0; 6.0]	4.0 [3.0; 5.0]	1.69	0.09
10 words memorizing test, *n*	4.0 [3.0; 5.0]	4.0 [3.0; 5.0]	−1.59	0.11
10 nonsense syllable memorizing test, *n*	3.0 [2.0; 3.0]	4.0 [2.0; 4.0]	0.81	0.42

### 3.2. EEG data

For the next stage of the analysis, patients who underwent simultaneous CABG + CEA were divided into two groups depending on the laterality of CA revascularization. The repeated measure ANOVA with a between-subject factor of GROUP (3 levels: CABG + left-sided CEA/CABG + right-sided CEA/isolated CABG), and within-subject factors of EXAMINATION TIME (2 levels: before/after surgery), ROI (2 levels: frontal and parietal), and LATERALITY (2 levels: left/right hemisphere) was performed for the indicators of theta1 rhythm power.

**Figure 3:**
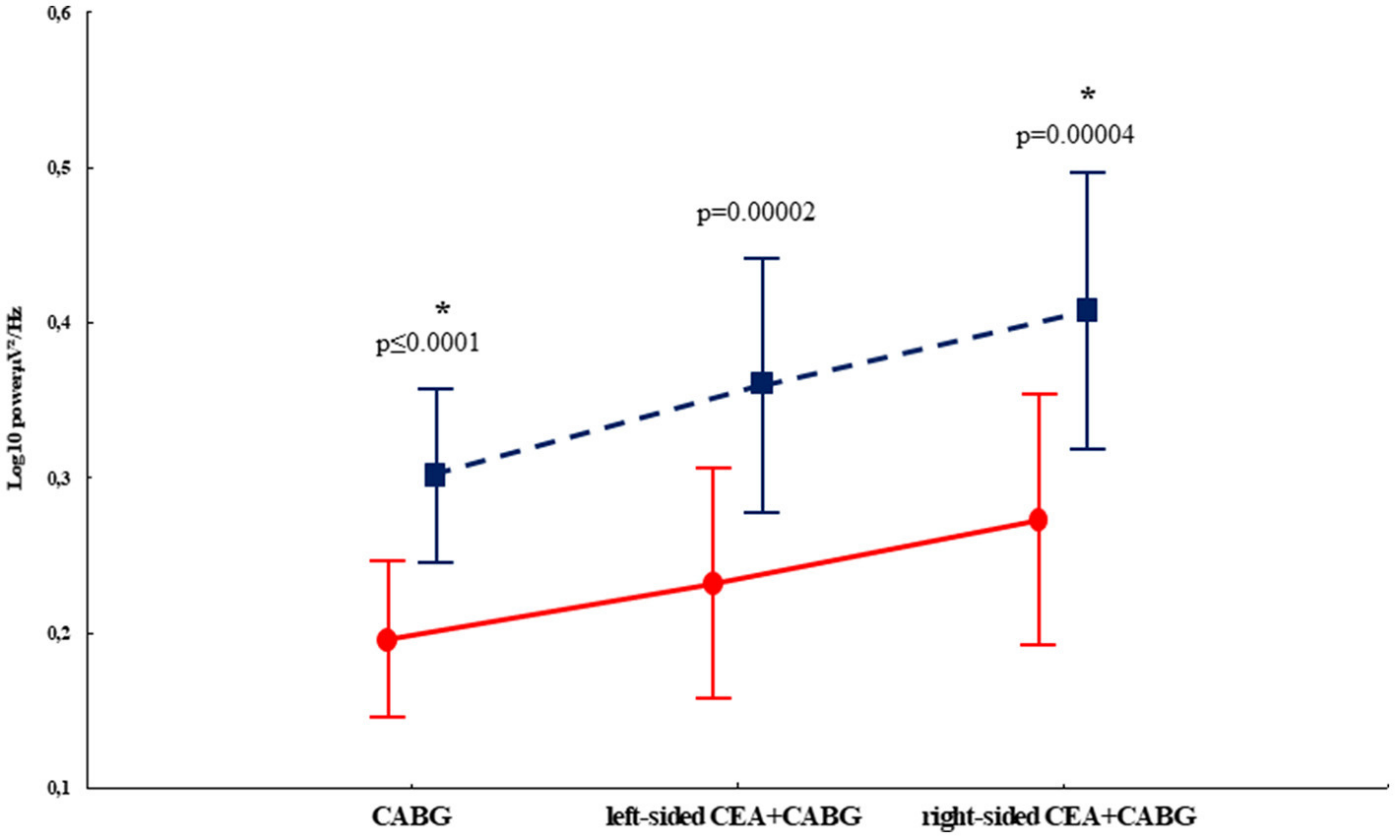
Postoperative theta1 rhythm power changes in the patients undergoing isolated CABG and simultaneous intervention (CABG + CEA). Solid red lines—the preoperative indicators, and dashed blue lines—the postoperative indicators. * indicates the significance level *p* ≤ 0.05 in the postoperative indicators of the right-sided CEA + CABG group in comparison to the CABG group.

The repeated measure ANOVA revealed the significance of the factor EXAMINATION TIME—F_(2,97)_ = 61.9, *p* ≤ 0.0001, η^2^ = 0.38. Theta1 power increase was noted at postoperative 7–10 days compared to baseline in both groups. As seen in Figure 3, the CABG + right-sided CEA group also differed from the isolated CABG group at 7–10 days after surgery (*p* = 0.04).

**Figure 4:**
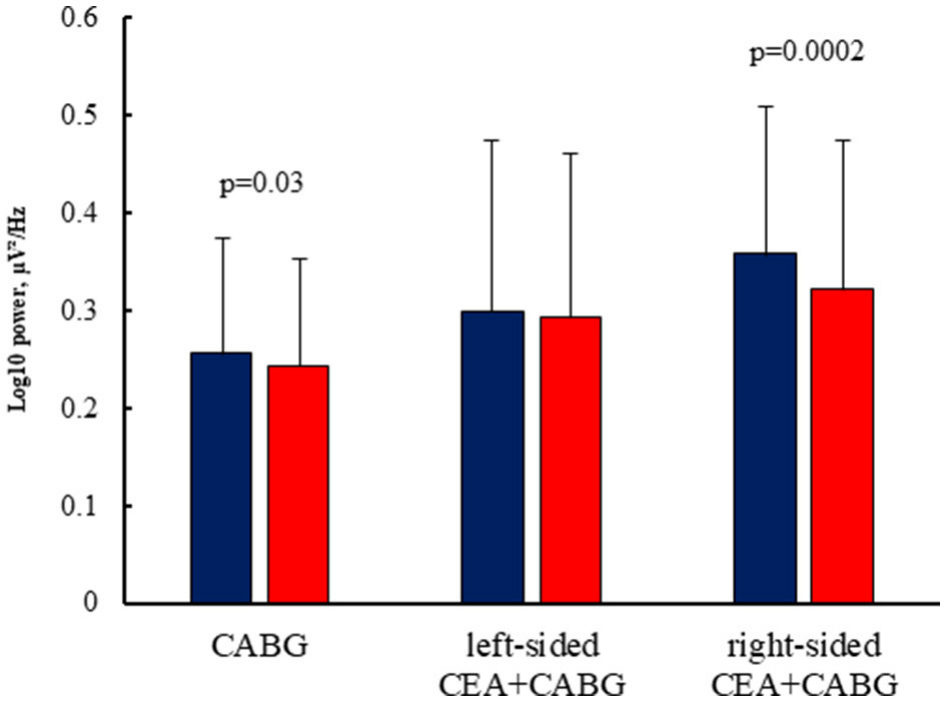
Lateral differences of theta1 power in the patients undergoing isolated CABG and simultaneous intervention (CABG + CEA). Blue columns—the power values in the left hemisphere, and red columns—the power values in the right hemisphere.

The interaction of factors GROUP × LATERALITY [F_(2,97)_ = 3.13, *p* = 0.047, η^2^ = 0.06] was deemed to be significant. The CABG + left-sided CEA group showed the least difference regarding the laterality of CEA‘s impact on theta1 power. Patients in the isolated CABG and the CABG + right-sided CEA groups had higher theta1 power values in the left hemisphere compared to the right hemisphere. This effect was more pronounced in the CABG + right-sided CEA patients (Figure 4).

**Figure 5:**
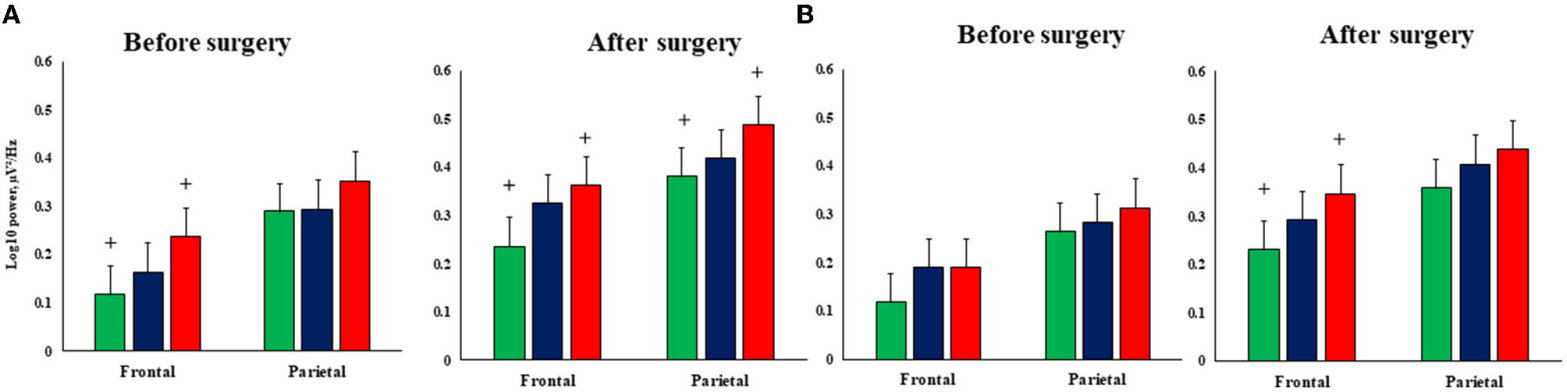
Topography of the postoperative theta1 rhythm power changes in the patients undergoing isolated CABG and simultaneous intervention (CABG + CEA). **(A)**—Left hemisphere, **(B)**—right hemisphere; green columns—isolated CABG, blue columns—left-sided CEA + CABG group, red columns—right-sided CEA+CABG group. ^**+**^ indicates the significance level *p* ≤ 0.05 in between-group differences.

The topography and severity of post-operative changes in the theta1 power rhythm differed in patients depending on the type of cardiac surgery. Thus, another significant interaction was revealed between the factors GROUP × EXAMINATION TIME × AREA × LATERALITY [F_(2,97)_ = 5.12, *p* = 0.008, η^2^ = 0.1]. The most pronounced theta1 power difference was found between the patients with isolated CABG and CABG + right-sided CEA. The CABG + right-sided CEA group had higher theta1 power values in the left frontal zone compared to patients with isolated CABG before and after surgery (Figure 5). After surgery, the CABG + right-sided CEA group also had higher theta1 power values in the left parietal zone and the right frontal zone compared to patients with isolated CABG, as seen in Figure 5.

## 4. Discussion

One of the main findings of this study was the lower cognitive performance (in terms of psychomotor speed and executive control) in patients with simultaneous intervention (CABG + CEA). It was noted before surgery and then it kept worsening in the early postoperative period at 7–10 postoperative days.

As stated in the review by Piegza et al. (2021), recent studies have highlighted the impact of impaired circulation due to high-grade carotid stenosis on cognitive deterioration. The study hypothesized that cerebral blood flow impairment could be an independent and potentially reversible factor determining cognitive decline in patients with severe stenosis (Lattanzi et al., 2018). Recent studies have also shown that aortic arch atheroembolism may play a critical role in brain injury and carotid disease and can serve as a marker of arch atherosclerosis, thus, increasing the risk of cardioembolism in CPB (Naylor et al., 2002).

Despite the fact that CABG + CEA patients had worse cognitive performance at baseline, the incidence of POCD was comparable in both simultaneous and isolated cardiac surgery groups. This can be explained by the prolonged CPB and operation time in isolated CABG patients. Another reason could be the recovery of cerebral circulation following carotid revascularization (Crespo Pimentel et al., 2022). In the study by Relander et al. (2022), CABG patients more frequently presented with short-term postoperative cognitive dysfunction compared to CEA patients. According to the authors, POCD is deemed to be a heterogeneous condition. It should also be noted that the incidence of POCD was estimated based on relative differences between the baseline and postoperative cognitive indicators. Thus, comparable POCD incidence in simultaneous and isolated cardiac surgery patients could be a manifestation of the ceiling effect that occurs when an independent factor (cardiac surgery) no longer has an effect on a dependent variable (cognitive performance).

The results of the EEG study have shown that the theta power increased in all patients in the early postoperative period compared to baseline regardless of the type of cardiac surgery. The increase in theta activity in resting-state EEG indicates cerebral dysfunction and may be a predictor of long-term cognitive impairment, meaning it is important for diagnostic purposes (Tarasova et al., 2018, 2019). In cardiac surgery patients, an increase in theta activity can be associated with cerebral ischemia during CPB. Cerebral atherosclerosis leads to endothelial dysfunction, perivascular nerve damage, arterial stiffness, and cerebrovascular insufficiency (de la Torre, 2017; Frey et al., 2018). Together, these adverse factors cause neuronal dysfunction, tissue atrophy, and damage in neural networks, resulting in cortical suppression by subcortical regions and the domination of low-frequency brain activity (Daulatzai, 2017).

Frontal and parieto-occipital brain zones are particularly vulnerable to cerebral hypoperfusion and microemboli associated with on-pump cardiac surgery (Pierik et al., 2019). The frontal and parietal zones are known as “cerebral watershed areas,” and they are perfused by the most distal branches of two major cerebral arteries (Momjian-Mayor and Baron, 2005; Amano et al., 2020). Furthermore, frontal brain zones play a key role in cognitive control and executive function (Widge et al., 2019; Friedman and Robbins, 2022). The results of our study showed significant intergroup differences in the theta power in the left and right frontal zones, in particular, worse executive function was noted in patients with simultaneous intervention and isolated CABG. Moreover, patients who underwent CABG + right-sided CEA demonstrated more pronounced theta power changes compared to patients with isolated CABG.

It has been recently reported that severe carotid stenosis can disturb the hemodynamic balance, illustrated by blood flow laterality (Zarrinkoob et al., 2021). In the study by Hedberg and Engström (2013), the authors showed that stroke occurs more frequently in the right hemisphere compared to the left hemisphere in the early postoperative period of cardiac surgery. As shown by the results of our study, contralateral stenosis of CA was observed in 76% of patients undergoing CABG + right-sided CEA. Thus, worse brain function in patients after CABG + right-sided CEA may be due to both surgical techniques focusing on right or left carotid arteries and cerebral blood flow impairment.

Summarizing the abovementioned points, the results of the study allow us to conclude that cardiac surgery with CPB is traumatic for the brain regardless of its features. Therefore, the search for diagnostic markers of the prediction of the impact of CABG and simultaneous intervention on brain function, and the implementation of the mandatory assessment of cognitive performance before surgery would be of great assistance to researchers. Further studies are necessary to identify the group of patients who will benefit the most from simultaneous revascularization. Moreover, we need more sensitive and specific neuropsychological tests that can assign each symptom to certain brain regions, as well as modern brain imaging techniques for diagnosis. For example, the sLORETA algorithm has been used to identify brain electrical patterns associated with various cognitive impairments in resting-state EEG.

## 5. Limitations

It is important to mention some of the limitations of this study. One limitation is a lack of data regarding cerebral blood flow laterality and the degree of the recruitment of collaterals. Another limitation of the study is the small sample of patients with simultaneous interventions. Moreover, the surgical procedure (anesthesia) time differed between the isolated CABG and the CABG + CEA group. Further research should be conducted to address these issues.

## 6. Conclusion

Thus, both CABG and simultaneous CEA and isolated CABG show comparable POCD incidence. It is important to note that all types of cardiac surgery resulted in the theta power increase in the early postoperative period compared to baseline. The CABG + right-sided CEA group is characterized by the most pronounced theta rhythm changes compared to patients undergoing isolated CABG and CABG + left-sided CEA due to a higher incidence of bilateral carotid artery stenosis and severe brain ischemia.

## Data availability statement

The data that support the findings of this study are not readily available because data sharing is not applicable. Further inquiries can be directed to the corresponding author.

## Ethics statement

The studies involving human participants were reviewed and approved by Ethics Committee of the State Research Institute for Complex Issues of Cardiovascular Diseases. The patients/participants provided their written informed consent to participate in this study.

## Author contributions

IT, OT, and DK: study concept, design, analysis, interpretation of data, and statistical analysis. IT, DK, OM, IS, IK, AS, and RT: data collection. OT, RT, and OB: critical revision of the manuscript and study supervision. IT: drafting of the manuscript. All authors contributed to the article and approved the submitted version.
